# Hyperthyroidism Presenting With Mania and Psychosis: A Case Report

**DOI:** 10.7759/cureus.22322

**Published:** 2022-02-17

**Authors:** Haris Asif, Ifediba Nwachukwu, Arshan Khan, Giovanna Rodriguez, Gul Bahtiyar

**Affiliations:** 1 Internal Medicine, Woodhull Medical Center, New York, USA; 2 Internal Medicine, Ascension St. John Hospital, Detroit, USA; 3 Endocrinology, Woodhull Medical Center, Brooklyn, USA

**Keywords:** hyperthyroidism, hyperthyroidism induced mania, hyperthyroidism induced psychosis, psychosis, secondary mania, mania

## Abstract

Graves' disease accounts for one of the most common causes of thyrotoxicosis. Most patients with Graves' disease present with classic signs and symptoms of hyperthyroidism. Psychosis and mood symptoms secondary to hyperthyroidism are rare. Here we report the case of a 37-year-old male with a history of Graves' disease with poor medication adherence who presented to the emergency department with psychotic features and hyperexcitability. He had excessive agitation, paranoia, and hyperactivity requiring restraints. He also endorsed insomnia and weight loss. He was admitted to the inpatient unit, and laboratory investigations were significant for a low thyroid-stimulating hormone, and elevated T3, T4, thyroid-stimulating antibodies, and thyroid peroxidase antibodies. The initial assessment was a primary psychiatric illness. The patient never had a personal or family history of psychiatric illness. Psychiatry and endocrinology were consulted for further recommendations. The patient was started on methimazole 30 mg, propranolol 100 mg, and hydrocortisone 100 mg, which resolved his symptoms.

## Introduction

Presentation of Graves’ disease depends on the age of onset, severity, duration of hyperthyroidism, sex, and comorbidities [1]. The most common symptoms of Graves’ disease include weight loss, fatigue, heat intolerance, tremor, palpitations, anxiety, disturbed sleep, and sweating [1]. On rare occasions, patients may present with psychosis [2], mania [3], or a combination of both as the initial presentation [4]. The first case of psychosis as a manifestation of thyrotoxicosis was reported over a century ago [5]. Since then, multiple cases have been reported in the literature. Although data are limited on the incidence of such presentations, these patients can be treated with anti-thyroid agents and beta-blockers [6]. Psychotropic medications are usually not indicated.

## Case presentation

A 37-year-old male with a past medical history of Graves’ disease was brought to the emergency department (ED) for excessive agitation for the past three weeks. As per his wife, the patient was not acting himself for the past few weeks. He had been more talkative, acting paranoid, hyperactive, and irritable. He would stay awake most of the night and had low frustration tolerance. Occasionally, he would laugh inappropriately. One year ago, the patient had similar issues and was diagnosed with Graves’ disease by his primary care physician. He was started on methimazole 10 mg and propranolol, which improved his symptoms, but he stopped his medications after three months of use due to non-adherence. As per his wife, the patient used marijuana only occasionally and did not have any baseline psychiatric problem. In the ED, vital signs were significant for a blood pressure of 160/95 mmhg. On further examination, he had cognitive slowing and seemed internally preoccupied and disorganized. Due to excessive agitation, the patient was restrained for a few hours. He was admitted to the hospital for further management. Further labs were significant for a thyroid-stimulating hormone level of <0.01 uIU/mL (reference range: 0.27-4.20 uIU/mL), free T4 level of 3.4 ng/dL (reference range: 0.9-1.8 ng/dL), free T3 level of 9.27 pg/mL (reference range: 1.80-4.60 pg/mL), thyroid-stimulating immunoglobulin level of 2.68 IU/L (reference range: 0.00-0.55 IU/L), and thyroid peroxidase antibody level of 344 IU/mL (reference range ≤ 34.9 IU/mL). Upon further consultation with endocrinology and psychiatry, the patient was started on methimazole 30 mg and propranolol 80 mg along with hydrocortisone 100 mg. After two days of treatment, the patient was calmer and more oriented. He was communicative and was back to his baseline as per his wife. He was discharged after almost five days of hospital stay.

## Discussion

Psychiatric symptoms have been described in thyrotoxicosis states. However, with the use of anti-thyroid medication, this has become an increasingly rare presentation. Basedow psychosis and myxedema madness have been described with thyrotoxicosis and hypothyroid state respectfully. Symptoms of irritability and hyperexcitability occur in a patient with Graves’ disease and rarely meet the criteria for a psychiatric diagnosis of mania. The exact mechanism of behavioral disturbance with thyrotoxicosis is unknown. However, it is suggested that the hyperthyroid-induced hyperadrenergic system disrupts the adrenergic pathway between locus coeruleus and the frontal lobe that subserve attention and vigilance [7]. There is a high prevalence of thyroid disorder in a patient with a primary psychiatric disorder. Further studies will be required to explore the complexity of this relationship.

Data are limited in terms of the epidemiology of psychiatric manifestation of Graves’ disease. According to the literature, the first case of manic psychosis in a patient with Graves’ disease was reported by Von Basedow [5]. In another study conducted in Christchurch, New Zealand, by Brownlie et al., 1% of all patients with thyrotoxicosis diagnosed over a 20-year period required psychiatric treatment. The majority of these patients were treated with antipsychotic medications as well as thionamides [6]. In another study conducted by Lo et al., out of the 22 patients with organic delusional disorders, four had psychosis caused by hyperthyroidism [8]. Data from the literature repeatedly proves that thyroid testing should be conducted in patients presenting with psychiatric symptoms in acute ED [9].

Treatment of a patient with thyrotoxicosis presenting with mania usually involves anti-thyroid medication and beta-blockers [10]. A psychiatric consultation is usually required to rule out the primary psychiatric diagnosis. Symptoms usually improve with treatment and lack of response points to an alternative diagnosis. Follow-up is recommended, and patient education is an essential part of the management of these individuals.

## Conclusions

Graves’ disease can present with psychosis, mania, or a combination of both. Treatment involves anti-thyroid medications and beta-blockers. Symptoms should improve with treatment. Lack of response should point to an alternative diagnosis.
